# Intravenous therapy device labeling in Intensive Care Units: an integrative review

**DOI:** 10.1590/0034-7167-2022-0049

**Published:** 2022-10-03

**Authors:** Geovane de Kassio Nunes, Juliana Faria Campos, Rafael Celestino da Silva

**Affiliations:** I Universidade Federal do Rio de Janeiro. Rio de Janeiro, Rio de Janeiro, Brazil

**Keywords:** Infusions, Intravenous, Drug Labeling, Critical Care, Patient Safety, Nursing., Infusiones Intravenosas, Etiquetado de Medicamentos, Cuidados Críticos, Seguridad del Paciente, Enfermería., Infusões Intravenosas, Rotulagem de Medicamentos, Cuidados Críticos, Segurança do Paciente, Enfermagem.

## Abstract

**Objectives:**

to synthesize the evidence on intravenous device labeling used to identify medications administered to patients in Intensive Care Units, with a view to preventing medication errors.

**Methods:**

an integrative review, in the LILACS, IBECS, Embase, MEDLINE, Scopus, Web of Science and CINAHL databases, from November to December 2021, using descriptors and selection criteria. Data were collected in 11 articles and subsequently classified, summarized and aggregated.

**Results:**

pre-designed labels, with pre-defined colors and information, help to prevent medication identification errors. There is still a lack of standardization in the practice of labeling syringes, intravenous lines, infusion pumps and saline solution bags. There are errors related to the lack of labeling devices or to their performance with incomplete information.

**Conclusions:**

device labeling is a barrier to defending the medication system safety and should be standardized.

## RESUMO

**Objetivos:**

sintetizar as evidências sobre a rotulagem de dispositivos intravenosos utilizada para a identificação de medicamentos administrados nos pacientes em Unidades de Terapia Intensiva, com vistas à prevenção dos erros de medicação.

**Métodos:**

revisão integrativa, nas bases de dados LILACS, IBECS, Embase, MEDLINE, Scopus, *Web of Science* e CINAHL, no período de novembro-dezembro de 2021, com uso de descritores e emprego de critérios de seleção. Os dados foram coletados em 11 artigos e, posteriormente, classificados, sumarizados e agregados.

**Resultados:**

rótulos pré-desenhados, com cores e informações pré-definidas contribuem para prevenir os erros de identificação dos medicamentos. Persiste a falta de padronização da prática de rotulagem em seringas, linhas intravenosas, bomba infusoras e bolsas de soro. Há erros relacionados à ausência de rotulagem dos dispositivos ou à sua realização com informações incompletas.

**Conclusões:**

a rotulagem dos dispositivos é uma barreira para a defesa da segurança do sistema de medicação, devendo ser padronizada.

## RESUMEN

**Objetivos:**

sintetizar las evidencias sobre el etiquetado de dispositivos intravenosos utilizados para identificar medicamentos administrados a pacientes en Unidades de Cuidados Intensivos, con el objetivo de prevenir errores de medicación.

**Métodos:**

revisión integradora, en las bases de datos LILACS, IBECS, Embase, MEDLINE, Scopus, *Web of Science* y CINAHL, de noviembre a diciembre de 2021, utilizando descriptores y criterios de selección. Los datos fueron recolectados en 11 artículos y posteriormente clasificados, resumidos y agregados.

**Resultados:**

etiquetas prediseñadas, con colores e información predefinidos, ayudan a prevenir errores de identificación de medicamentos. Todavía hay una falta de estandarización en la práctica de etiquetado de jeringas, líneas intravenosas, bombas de infusión y bolsas de suero. Hay errores relacionados con la falta de etiquetado de los dispositivos o con su realización con información incompleta.

**Conclusiones:**

el etiquetado de los dispositivos es una barrera para defender la seguridad del sistema de medicamentos y debe ser estandarizado.

## INTRODUCTION

Among healthcare-associated incidents, those involving medication therapy are common in institutions, likely to occur at all stages of the medication system, from prescribing, dispensing, preparing and administering the medication to monitoring, with possibilities of serious harm and even death of patients^(1-2)^.

These medication incidents that cause harm to patients are called adverse medication events, which have medication errors as one of their causes^(1)^. Medication errors are any preventable events that can cause or induce medication misuse and even harm while the medication is under the control of the patient, health care provider, or consumer^(3)^.

In 2017, the World Health Organization reinforced its concern about medication incidents with the launch of the 3^rd^ Global Patient Safety Challenge - Medication Without Harm, whose goal is to reduce serious and preventable medication-related harm by 50% by the year from 2022^(1)^.

Medication errors are considered a public health concern, being present in various care scenarios^(4-6)^. In the context of Intensive Care Units (ICUs), in particular, such a problem may be even greater, since they are units that receive patients with acute and severe clinical situations^(7)^. They are patients who depend on multiple medications administered concomitantly aiming at maintaining the hemodynamic balance of their clinical status, especially through intravenous (IV) medication administration. Therefore, due to the critical nature of their diseases and the great need for medication interventions, the occurrence of medication errors in this group can have very serious consequences^(7)^.

In the specificity of errors involving IV medications administered to ICU patients, we highlight, in this research, those related to identification, which involve the identification of syringes, infusion pumps, saline solution bags, IV routes and lines. The absence, incompleteness and/or misidentification of these devices can cause changes in the infused dosage, change in medication administration, change in IV bolus solution, among other possibilities, with potential risks to patient safety.

An example of these errors is seen in the cases of failures in the identification of routes and IV lines. Multiple IV medications given to critically ill patients lead to more IV bags, infusion pumps and IV lines. This situation causes visual disarray and intertwining of IV lines, making it difficult to quickly and correctly identify what is being infused, especially in situations of complications. Thus, the chances of errors in the handling of IV lines increase, an aspect that has been pointed out by the literature^(8-9)^.

A study conducted with 40 nurses in a simulated ICU setting aimed to identify the risks associated with multiple IV infusions and the ability to administer them safely. Among the findings, 7.7% of errors were identified in the screening of IV lines during the identification of infusions^(8)^.

Research reviewed the reporting reports of medication errors in Pennsylvania from 2004 to 2013. In this analysis, 907 errors related to IV-line handling were identified. The most common mistakes were the inclusion of setting infusion rates, changes when handling disordered IV lines, and IV lines not connected to patients. Potentially dangerous medications were involved in 71% of errors associated with IV lines, and heparin was the most commonly found medication, an error that can lead patients to death^(9)^.

Given this scenario, identification errors can be minimized with the adoption of barriers that contribute to the medication and route identification, such as label/labeling use. The term label is defined as the display of written, printed, or graphic material in a container. Labeling is defined as all labels and other printed, written or graphic materials on any article or any container^(10)^.

The term medication labeling is linked to using written, printed or graphic materials in a medication container or packaging, and may include effects, duration, dose, routes, warnings, among other relevant information^(11)^. Thus, it is understood in this research that medication labeling refers to label content and type of material used in devices used to administer a particular medication, such as syringes, saline solution bags, bottles, infusion pumps and IV lines

Device labeling emerged in the area of anesthesia, created by the International Society of Anesthesia and, currently, different shapes and colors associated with the medication class are used, in addition to visibly adequate texts and sources^(12)^.

The International Organization for Standardization (ISO) 26825 publication was considered a milestone regarding labeling practices, as it established guidelines for standardizing this care. However, the publication of this standard is aimed at the anesthetic area and the syringe device. Although its use can be extended to other areas, such as intensive care, and to other devices, this is not a practice validated by the regulatory agencies themselves. Thus, labeling practices vary throughout the world and are commonly established by the hospital and/or professional institution itself, which impacts the way medications are identified in their devices^(13-14)^.

In Brazil, the recommendations regarding labeling refer to medication bottles^(15)^, however, in the case of lines IV, the researchers’ practical experience shows that these are still frequently identified with handwritten, non-standardized adhesive tapes, with differences according to institutional policies.

Based on the above, it is assumed that labeling has the potential to promote numerous benefits for the team and patients, such as: rapid and correct identification of IV infusion; reduced connection errors between syringes and IV lines; and signaling of an exclusive route for medications considered to be of high risk, in order to avoid handling or performing inappropriate flushing. Given the need to understand how labeling has been used in ICU settings, with a view to thinking about strategies for the prevention of medication errors that can cause irreversible damage to patients, this review is justified.

## OBJECTIVES

To synthesize the evidence on intravenous device labeling used to identify medications administered to patients in Intensive Care Units, with a view to preventing medication errors.

## METHODS

### Study design

This is an integrative literature review, a research method that comprises the search, critical assessment and synthesis of relevant research on a given topic^(16)^. The methodological approach adopted for developing this review was composed of six phases^(16)^, namely: theme identification and hypothesis or research question selection; establishment of inclusion and exclusion criteria for studies; definition of the information that would be extracted from the included studies/study categorization; assessment of studies included in the integrative review; interpretation of results; and review report. The PRISMA tool was used to support the presentation of this review.

### Methodological procedures

After identifying the focus theme of this review, in the first phase of the method, the review question, the descriptors and the bases that served as a source of search for scientific articles were defined. The guiding question was structured through the mnemonic strategy PCC (Problem, Concept and Context), proposed by Joanna Briggs Institute^(17)^, in which: P (medication errors); C (medication labeling); C (critical care), which gave rise to the following question: what is the evidence on labeling IV therapy devices for medication identification and prevention of medication errors in the ICU? This question guided the choice of descriptors and keywords referred to in each language (Portuguese, English and Spanish), which were defined as shown in Chart 1.

**Chart 1:** Descriptors adopted to carry out search strategies

Descriptors			
**Type**	**Problem**	**Concept**	**Context**
**DeCS**	“*Segurança do Paciente*” OR “*Prevenção de Acidentes*” OR “*Erros Médicos*” OR “*Erros de Medicação*”	“*Rotulagem de Medicamentos*”	“*Cuidados Críticos*” OR “*Enfermagem de Cuidados Críticos*”
**MeSH**	“Patient Safety” OR “Accident Prevention” OR “Medical Errors” OR “Medication Errors”	“Drug Labeling”	“Critical Care” OR “Critical Care Nursing”
**CINAHL Subject Headings**	“Patient Safety” OR “Adverse Drug Event” OR “Medication Errors” OR “Health Care Errors” OR “Treatment Errors”	“Drug Labeling” OR “Product Labeling”	“Critical Care” OR “Critical Care Nursing” OR “Intensive Care Units”
**Emtree**	‘Patient safety’/de OR ‘Accident Prevention’/de OR ‘Medical Error’/de OR ‘Medication Error’/de OR ‘Potentially Inappropriate Medication’/de	‘Drug Labeling’/de	‘Intensive Care’/de OR ‘Intensive Care Nursing’/de

The chosen databases were Latin American and Caribbean Literature (LILACS), National Library of Medication (MEDLINE via PubMed), Cumulative Index to Nursing and Allied Health Literature (CINAHL), Spanish Bibliographic Index of Health Sciences (IBECS), SciVerse Scopus (Scopus), Embase and Web of Science. The data search was carried out from November 1 to December 31, 2021, through online and direct access to each database via the CAPES portal.

Search strategies in databases that had vocabulary control, such as LILACS, MEDLINE, CINAHL and Embase, composed a cross of indexed and standardized descriptors in each base, plus additional keywords customized for the target language. In the IBECS, Scopus and Web of Science databases, due to the fact that the descriptors applied in the other databases are not standardized for these, it was decided to carry out the crossings using only keywords already standardized in their indexes, which were chosen according to the PCC strategy adopted.

In compliance with the PRISMA tool recommendation, Chart 2 details the strategy of crossing the terms used in one of the databases (MEDLINE), as an example of the methodological procedures adopted to capture the articles in the research.

**Chart 2:** Example of search strategy applied in the MEDLINE database

(“Patient Safety”[MeSH Terms] OR “Accident Prevention”[MeSH Terms] OR “Medical Errors”[MeSH Terms] OR “Medication Errors”[MeSH Terms] OR safety[Title/Abstract] OR error[Title/Abstract] OR errors[Title/Abstract] OR “adverse event”[Title/Abstract] OR “adverse events”[Title/Abstract]) AND (“Drug Labeling”[MeSH Terms] OR ((label[Title/Abstract] OR labels[Title/Abstract] OR labeling[Title/Abstract] OR labelling[Title/Abstract] OR sticker[Title/Abstract] OR stickers[Title/Abstract] OR adhesive[Title/Abstract] OR adhesives[Title/Abstract]) AND (drugs[Title/Abstract] OR drug[Title/Abstract] OR medication[Title/Abstract] OR medications[Title/Abstract] OR medicines[Title/Abstract] OR medicine[Title/Abstract]))) AND (“Critical Care”[MeSH Terms] OR “Critical Care Nursing”[MeSH Terms] OR “intensive care”[Title/Abstract] OR “intensive unit”[Title/Abstract] OR “critical care”[Title/Abstract] OR “critical unit”[Title/Abstract])

The second phase was to outline the criteria for selecting the research corpus and its application in the research process. Thus, the following inclusion criteria were adopted: scientific articles, results of primary studies; published in English, Portuguese, or Spanish; period from 2008 to 2021, time frame established by the start of publication of guidelines on labeling of devices IV; full text available in selected databases; refer to labeling IV devices in ICU settings. Articles that did not establish links with patient safety were excluded.

In the selection process, after crossing the descriptors and keywords in each chosen base, initial filters of full text, article design and language were applied. Then, with the help of Mendeley, duplicate references were excluded. After this step, the articles found were first submitted to an exploratory reading of title and/or abstract. Those that did not comply with the theme were pre-selected for the reading phase in full. In the full text assessment, articles were assessed regarding their eligibility considering the answer to the research question. This phase of article selection was carried out by two researchers, with disagreements about the final inclusion of the article being decided jointly.

### Dara collection, organization and analysis

Data collection in the articles corresponded to the third step of the method, which was based on an instrument previously prepared by the authors that contained the information defined for data extraction, such as identification of author and article, objective, methodological characteristics and results, in addition to a section for assessment regarding clarity and scope in relation to the study theme. These data, after collected, were summarized in a table that gathers the main information related to the research question. The selected studies received the letter “S”, for Study, followed by the Arabic number.

In the fourth phase, the critical analysis of included studies took place, taking into account the problem and the research question outlined. Thus, the previously organized data were categorized into evidence synthesis units, in which the data were aggregated and the desired synthesis of knowledge was obtained. Such synthesis units make it possible to apprehend evidence of IV device labeling practice by professionals in intensive care, their links with patient safety, the types/characteristics of labels and their effects on the prevention of medication errors.

After presenting the results regarding this synthesis, they were interpreted in the fifth phase, considering the existing literature on the subject and the concepts that support patient safety, which resulted in the final product of this integrative review, the sixth phase of the method.

## RESULTS

**Figure 1:**
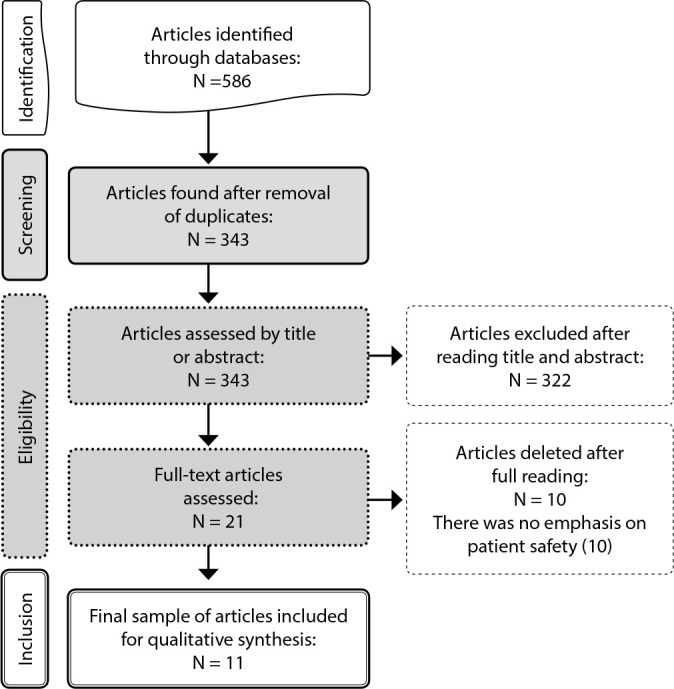
Article selection flowchart

This study consisted of a sample of 11 articles. Figure 1 shows the article selection flowchart produced by the authors. Regarding the origin of scientific productions, 27% of studies (three articles) were developed in Canada and 27% in Brazil. The other countries had one article each: Germany (9%), Australia (9%), United Kingdom (9%), United States (9%) and Malaysia (9%). Regarding the year of publication, three articles (27%) were published in 2016, followed by 2019, 2012 and 2008, with two articles published in each of these years (18%). Finally, there was only one article (9%) for each of the following years: 2018 and 2014.

The selected articles were organized in Chart 3, which presents the synthesis of the relevant findings for the study.

**Chart 3:** Synthesis of selected articles on intravenous device labeling

	Title/country/year	Objective	Design	Conclusion
S1^(13)^	*Avaliação da rotulagem com código de cores para identificação de medicamentos intravenosos* Brazil/2019	Analyze the opinion of nursing professionals about the design, practicality of use and usefulness of color-coded labeling in a pediatric ICU.	Cross-sectional, quantitative Total number of participants: 42	The labeling technology was considered appropriate for the design and practicality for all devices, as well as useful in preventing medication errors, reducing the average time developing labeling tasks.
S2^(18)^	Untangling Infusion Confusion: a comparative evaluation of interventions in a simulated intensive care setting Canada/2019	Assess the impact of interventions performed by participants in four different conditions in a simulated ICU scenario: current practice; using IV-line labels and infusion organizers; with infusion pump; and with an infusion route lighting system.	Experimental, quantitative Each participant should perform two tasks in each condition: correctly identify and disconnect an infusion. Total number of participants: 40	Using IV-line labels and infusion organizers may increase accuracy and efficacy in the correct identification of IV medication infusion.
S3^(12)^	Standardized drug labelling in intensive care: results of an international survey among ESICM members USA/2009	Compare a color-coded label for high-risk medications with the current labeling practice in a simulated ICU scenario.	Quasi-experimental, quantitative Total number of participants: 61	Using color code improved identifying bags and IV lines, facilitated the identification of errors and decreased the average time in tasks. The task of color-coding the syringe did not show a significant difference when compared to the control scenario, as participants reported difficulty in handling syringe labels.
S4^(19)^	Standardized medication labelling in intensive care: results of an international survey among ESICM members Germany/2012	Investigate whether standardized medication syringe labeling is used in ICUs, whether these standards are similar in other countries, and whether intensivists expect standardized medication syringe labeling to be provided by the pharmaceutical industry.	Survey type, multinational, quantitative Total number of participants: 482	The adoption of standardized syringe labeling in terms of color, design and structure is still lacking among many ICUs, leading to variability in labeling policies between sectors within the same hospital and between hospitals in the same region. This failure can induce professionals to misidentify medications.
S5^(14)^	Multiple Intravenous Infusions Phase 2a: Ontario Survey Canada/2014	Investigate practices or policies that can help identify and prevent risks in patient safety.	Prospective, quantitative study Total number of participants: 64	Inconsistencies between policies and practices were found in some units. It was concluded that these non-conformities in label use can lead to erroneous handling of continuous infusions, erroneous disconnection of infusion, difficulty in administering medications through IV lines in the scenario of clinical emergencies.
S6^(20)^	Variability of intravenous medication preparation in Australian and New Zealand Intensive Care Units Australia/2016	Examine variability in the preparation of continuous infusion medications.	Survey-type study, quantitative Participants: 40	There was little variability in device labeling among the investigated ICUs. Labeling, whether for syringes or medication bags, is color-coded and the medication name is handwritten.
S7^(21)^	Variability in the concentrations of intravenous medication infusions prepared in a critical care unit The United Kingdom/2008	Check if there is a relationship between syringe label quality and medication preparation.	Audit study N=149 syringes The discarded syringe labels received a score on a scale of 11 points for quality, as well as the analysis of the concentration of residual solutions present in the syringes.	Better labeled syringes were more likely to contain the medication at the correct concentration.
S8^(22)^	Errors associated with IV infusions in critical care Canada/2012	Determine the most frequent type of error with IV medications.	Observational, prospective, quantitative study N=1,882 infusions	Incomplete labeling of IV lines was the most common error and posed a risk to patient safety.
S9^(23)^	An investigation of errors: the preparation and administration of parenteral medications in an intensive care unit of a tertiary teaching hospital in Malaysia Malaysia/2015	Investigate type, incidence and factors related to errors with IV medications.	Prospective observational study N= 122 IV medications prepared by 39 nurses	Not labeling the syringe is supposed to be an indicator for medication preparation failure, with more chances of dose errors.
S10^(24)^	*Erros no preparo de medicação intravenosa em uma unidade de terapia intensiva neonatal* Brazil/2018	Analyze the occurrence of errors in IV medication preparation in the neonatal ICU.	Observational, quantitative study N= 70 medication doses	Labeling has not been commonly used in care practice.
S11^(25)^	*Utilização de barreiras de segurança no preparo de drogas vasoativas e sedativos/analgésicos em terapia intensiva pediátrica* Brazil/2018	Analyze safety barrier use in vasoactive medication and sedative/analgesic preparation in a pediatric ICU.	Observational, quantitative study N=17 professionals in 204 observations on medication preparation	Labeling practice has been carried out incompletely.

The synthesis constructed after the aggregation of the selected research results indicated that the evidence on IV device labeling was organized into three units.

### Label types and effects on prevention of medication errors in intensive care

The data from this synthesis unit provide indications of the potential benefits of the characteristics of labels for IV lines, syringes, infusion pumps and IV bags in preventing medication errors. Among the impacts of using labeling evidenced in the studies, in S1^(13)^ and S2^(18)^, using color coding for IV lines helped to distinguish which medications were infused, increasing effectiveness in correctly identifying medication infusion and providing greater ease in handling multiple infusion pumps and lines. Using color code also improved the identification of bags, reducing the average time developing labeling tasks, facilitating the identification of errors and reducing the average time of tasks in general (S1^(13)^, S3^(12)^).

Moreover, in S2^(18)^, participants who used electronic medication identification through the medication library of infusion pumps required more time to handle IV lines when compared to participants who handled pumps in common use, in association with using labels on IV lines.

Regarding the characteristics of label types, it is noteworthy that, in S1^(13)^ and S3^(12)^, the labels were differentiated by color codes according to the pharmacological class, according to the ISO 26825 standard, being used for IV-line syringes, infusion pumps and saline solution bags. In S1^(13)^, writing the medication name was differentiated using CAPITAL LETTER when names were similar. Specific labels for potentially dangerous medications have also been standardized with white background and red letters. In both S1^(13)^ and S3^(12)^, the label was printed with the medication name, and the other information was filled in manually.

In S2^(18)^, the IV-line label did not use colors for pharmacological classes, but used a white background in its manufacture. The label was adhesive and printed with the medication name, with visualization on both sides of the device, and fixation occurred 8 cm below the continuous infusion pumps and at the end of each IV line.

In S7^(21)^, discarded medication labels were analyzed (midazolam, insulin, magnesium, norepinephrine), measuring the concentration of residual solutions present in the syringes. The syringe label should contain the medication name, dose, concentration, diluent, patient name and location, initials of the person who prepared it, initials, day and time. Of 149 syringes, 61 were correctly labeled; 51% of labels did not have the medication concentration; and 47.6% had no information about the diluent. From the analysis of the 149 discarded syringes, the authors found that the best labeled syringes were more likely to contain the medication at the correct concentration. Magnesium syringes were the least likely to be labeled correctly, and insulin syringes were the most correctly labeled.

In S9^(23)^, the results were similar to those of S7^(21)^: in 15% of the doses observed, the syringes were not labeled correctly. The syringe label should contain the medication name and dose information. Poorly labeled syringes had more errors per dose (1.9 times more) when compared to well-labeled syringes (1.1 times). The authors attributed these rates to the absence of a specific label for the syringe, since, in the scenario studied, there were only pre-printed labels for bags.

### Medication identification errors in labeling intravenous devices by professionals in the Intensive Care Unit

It was found that, in ICU settings, professionals make medication errors related to their labeling practice, when they do not label a device or when they label it incompletely, without identifying the medication route, concentration, dilution, name. These situations were considered in the studies as medication errors.

In S10^(24)^, of 70 doses observed that were prepared by nursing professionals, 65.7% did not receive any label on the devices in which they were inserted. Among those containing the label, only 11.4% were complete. This absence of information filled out by professionals on labels was a practice commonly observed in other studies, such as S5^(14)^, S8^(22)^ and S11^(25)^.

In S11^(25)^, for instance, of the 204 medications administered by saline solution bags, 12.2% were not labeled by professionals. Identification with patients’ full name was present in only 3.4% of labels; 99.4% of devices were labeled without a route description; the label was fixed on only 17.3% of medications and, even so, there was no compliance regarding the fixation place.

These results were in line with those obtained in S5^(14)^ and S8^(22)^. In S8^(22)^, 5,641 errors were identified. Incomplete labeling of IV lines comprised 31.5% of all errors, and incorrect labeling, 26.8%, representing a risk to patient safety. Discrepancies in labeling IV lines were more common during day shifts. Finally, in S5^(14)^, 95% of participants reported not performing IV-line labeling. There were disagreements about how these labels should be filled and exchanged. Some participants reported that some labels were too small for use.

### Variability in labeling practice for the identification of devices in the Intensive Care Unit

Overall, there was great variability in studies regarding the label structure, which could be printed labels (S2^(18)^ and S5^(14)^), with information related to the previously printed medication; preprinted (S1^(13)^, S3^(12)^, S5^(14)^ and S11^(25)^), previously printed content, with space to fill in other information manually; and completion only handwritten (S5^(14)^ and S6^(20)^).

As for the label content for IV devices, the information that appeared most frequently was the medication name in seven studies and the dose in three studies. Labels that contained more information were the syringes and saline solution bags, mainly the medication name, dose, concentration, diluent, patient name and location, initials of the person who prepared it, initials, day and time. IV line and infusion pump labels presented less information, with emphasis on the medication name and route (central or peripheral), in the case of the lines. Infusion pump labels were found only in two articles, containing the medication name, infusion dose/volume and color related to the medication’s therapeutic class according to ISO 26825^(13-14)^.

Regarding the evidence about lack of standardization, in S5^(14)^, 29.6% of participants mentioned that there is no standard practice where they work in relation to IV-line labeling. This result was repeated in S4^(19)^ in relation to syringes, when only 39% reported that standardized medication labeling on the syringe was used in the ICU where they work and 30% of participants reported using ISO 26825 (in its original form). In S6^(20)^, there was a lower degree of variability in labeling practice among the ICUs of the institutions studied in Australia and New Zealand, particularly for syringes and saline solution bags, which should be made with color code and with the medication name handwritten.

## DISCUSSION

The results found on labeling IV therapy devices in the ICU indicated: absence of a uniform pattern of structuring the labels in terms of content; color and design applied to different infusion devices; positive effects of labeling on patient safety and on better performance of activities by nursing professionals, especially studies that used pre-designed labels, with pre-defined colors and information or with spaces to be filled in handwritten form; and low compliance with IV device labeling (saline solution bags, syringes and IV lines) or their performance with incomplete information by nursing professionals, aspects that are configured in medication identification errors.

Regarding this last result related to non-labeling or incomplete labeling of IV devices, it is pointed out that, in critical patient care environments, using potentially dangerous medications is frequent, which are recognized for their high chances of causing harm to patients when there is an error in their use.

On this, a systematic review developed to determine the prevalence of errors and the main damage caused by using potentially dangerous medications found an overall prevalence of 16.3%, of which 0.01 led to patient death. The highest prevalence of damage occurred after using medications such as potassium chloride, insulin and epoprostenol^(26)^. Already analysis of incident reports involving medications identified that the potentially dangerous were involved in 281 of 624 events, i.e., responsible for 45% of security failures^(27)^.

Errors related to the handling of IV lines of potentially dangerous medications, for instance, are considered a problem of great relevance. This relevance is seen in the list of the top ten health technology-related hazards drawn up by the Emergency Care Research Institute (ECRI) for 2015. Among them, errors with IV lines were cited, which lead to incorrect medication and solution administration, mainly due to the tangle of lines that exist when multiple IV infusions are administered to a single patient. Among the risks involved in handling IV lines, the infusion line being connected to the wrong device, delivering inappropriate fluid to patients, and doses being titrated wrongly or through an inappropriate route stood out^(28)^.

One of the situations mentioned in this list is the notification of an incident, in which there was IV medication administered in an epidural catheter. Thus, difficulties in discerning one IV line from another and the tangle of lines can compromise correct tracking from line to IV pouch and vice versa. Therefore, to prevent these errors, it is recommended to label each infusion with the medication name being infused^(28)^.

This context indicates that nursing care for critically ill patients with multiple IV infusions is complex, with a substantial risk of errors in the identification of IV medications, which directly interfere with patients’ recovery, ranging from minor clinical repercussions to cases of death. Therefore, the importance of complying with using labels in care practice is reiterated, promoting a safer medication system.

As for the label characteristics and their effects on the prevention of medication errors, the results obtained can be compared with those of studies developed in other settings where intensive care is also provided, such as the operating room and emergency room. These results reiterate that characteristics, such as pre-design of labels and color, are important for the correct identification of medications, increasing practicality, reducing the average time spent on labeling tasks, in addition to identifying errors more quickly.

Research developed in a simulated scenario sought to quantify the impact of medication label design on safety. To this end, 96 anesthetists were randomly divided into two groups, with the objective of identifying the hetastarch bottle in the anesthesia cart of a simulated operating room, to fulfill a medical prescription for hemodynamic instability correction. The anesthesia cart contained three 500 ml bottles of hetarstarch and one 500 ml bottle of lidocaine. In the intervention situation, the medication bottle should be identified using the redesigned label; in the control situation, using the normal label. The percentage of participants who correctly selected the hetastarch bag after adopting the new medication label was significantly higher than with the current model (63% versus 40%). The authors concluded that using a new label helped participants select the correct medication and avoid harm to patients^(29)^.

Also in the operating room, it is verified that the studies bring data mainly on syringe labeling. One of them compared medication errors before and after the implementation of a secure medication bundle and a barcode-based computerized medication system. Among the measures adopted in the bundle were syringe labeling and color use. A barcode system was also used that generated a label for each syringe. Errors with syringe change were significantly reduced after implementing the bundle. After using a barcode, there was no difference in error index. Evidence from this study suggested that identifying the syringe with a label is associated with a reduction in medication errors from needle exchanges^(30)^.

A study in a pediatric resuscitation scenario in an emergency compared syringes previously prepared with medications and labeled with color codes with the syringes used in conventional medication administration. The group with the conventional syringe had an average time of 47 seconds to administer the medication. Of the 118 doses administered, 17% had critical dosing errors. The group with the pre-filled and color-labeled syringe took an average of 19 seconds, and of the 123 doses administered, there were no medication errors. It was concluded that labeled prefilled syringes shortened the time to administer the medication, as well as reduced critical dosing errors in pediatric cardiac arrest^(31)^.

The international studies that composed the analyzed corpus indicated that there is still variability in labeling practice regarding the label structure and content. Particularly, the Brazilian National Health Regulatory Agency (*Agência Nacional de Vigilância Sanitária*) recommends identifying medication containers with patient name, date of birth, medication, time and route of administration, dose and flow rate in the case of infusions. Route identification aims at correct connection during IV medication administration. As for continuous IV infusions, no particular label is indicated, only to conference drip speed and programming^(32)^.

There are also recommendations regarding label use on potentially dangerous medications in the storage stage, as mentioned: “the use of colored labels in the storage process with the writing of high-risk medication”. However, recommendations on labels in the administration phase are absent. Furthermore, according to the protocol for safe medication administration, medications with similar spellings and names must have part of the name written in bold and capital letter^(32)^.

It is also noteworthy that the Institute for Safe Practices in Medication Use - Brazil (*Instituto para Práticas Seguras no Uso de Medicamentos - Brasil*) presented a set of recommendations for the prevention of errors involving potentially dangerous medications. Among these, we emphasize: ensure the correct label of the syringe wit patient name, solution, concentration and route of administration; identify pouches of vinca alkaloid solutions labeled “Intravenously only - fatal if administered otherwise”; use labels that emphasize the difference in spelling with capital letter and in bold^(33)^.

Considering the results, it is considered that standardized labeling of devices should be encouraged and rethought, so that it occurs in the daily work of nursing in a safe way, since these professionals are responsible for medication preparation in the ICU. This strategy can contribute to: modifying problems related to low compliance with labeling in intensive care settings, which leads to lack of information or transcription errors; avoid errors related to handling IV lines, changing syringes or IV bags; and provide greater safety of medication administration in emergency situations.

The evidence presented in this research on labeling IV devices reiterated the importance of this practice for patient safety, i.e., when performed properly, it contributes to reducing medication errors and increasing professional safety in providing nursing care. Thus, this practice can be considered an important barrier to defending the medication system safety.

From the conceptual perspective of safety, the weaknesses of the health system (failures) can be compared to the holes in a Swiss cheese, in which, for an incident to happen, the alignment between the holes in the cheese must occur. In view of this, we seek to propose barriers that prevent this alignment of the holes and that the damage affects patients^(15)^.

Therefore, based on the understanding that labeling improves the identification of information about the medication and visually indicates the pharmacological class to which it belongs, it can be configured as the last barrier before administering a medication. It alone does not guarantee that a medication is prepared correctly, as if a medication has been prepared incorrectly and labelled correctly, the label will not fit what will actually be administered. But if a medication has been prepared correctly and mislabeled, unlabeled or with incorrect information, it may be administered to another patient as well as there may be underdosage or overdose.

An example of these situations is seen in the research that, from the errors identified in the handling of IV lines of potentially dangerous medications, presented three real cases: a) a patient was using fentanyl and was requested for a bolus of physiological saline solution, however, the saline and fentanyl lines were mixed up and a bolus of fentanyl was given instead of saline; b) tracing the IV lines of heparin and nitroglycerin caused nitroglycerin to be administered at 30 ml/h, when, in fact, it should be set at 1.5 ml/h. Heparin was infused at 1.5 ml/h instead of 30 ml/h as a consequence of the error; and c) phenylephrine bag was on the line in which IV fluid was programmed at 150 ml/h, leading patients to death^(9)^.

### Study limitations

The limitation of this study was related to the lack of detail in some studies of information on the characteristics of the labels used in the studies, which limited the in-depth analysis of the findings in the synthesis of knowledge presented in this review.

### Contributions to nursing and health

Medication errors are currently the most common causes of morbidity and mortality in patients in the context of patient safety. In turn, the costs related to these errors represent an important burden on the health system and an important source of waste^(1,34)^. This reinforces the need for preventive measures that envision effective impacts on patient safety at all stages of the medication system and that maximize the offer to patients of care permeated by safe actions.

The synthesis of knowledge obtained in this study about labeling IV therapy devices in ICU settings contributes to the development of actions aimed at creating barriers to prevent medication errors related to the identification of devices for medication administration, as well as to reduce the costs associated with them, thus bringing potential benefits to patient safety.

## CONCLUSIONS

The results indicated that pre-designed labels, with colors and predefined information for bags of saline solution, IV lines, infusion pumps and syringes, contribute to prevent errors in medication identification. Moreover, they showed that well-labeled syringes had lower chances of medication dosing errors. Despite these effects on patient safety, there is still a lack of standardization of labeling practice to identify medications in syringes, infusion pumps, IV lines and IV bags in institutions, both in terms of content, structure and color. Additionally, it was found that there is a low compliance with labeling IV devices or its performance with incomplete information in practice by nursing professionals, which configured an error in the identification of medications.

Based on these results, further studies are recommended to develop proposals for labels to identify medication delivery devices and to test their applicability in practice, in order to adopt a standard of information visualization. Standardization of labels across institutions can lead to faster and more effective patient interventions in administering, monitoring, and removing IV infusions in critically ill patients. In addition to this, it can contribute to the nursing team’s work in the correct identification of these infusions and, thus, minimize medication errors, such as connections and disconnections of infusions, flushing in wrong routes, among others. In this way, both patients benefit, when receiving medication therapy without interruptions and errors, and professionals, when they feel safer in the handling of such devices.
